# ASO Author Reflections: Contemporary Trends in Referral Surgical Cancer Care

**DOI:** 10.1245/s10434-025-17173-4

**Published:** 2025-03-18

**Authors:** Kelsey B. Montgomery, Kristy K. Broman

**Affiliations:** 1 Department of Surgery, University of Alabama at Birmingham, Birmingham, AL USA; 2 Institute for Cancer Outcomes and Survivorship, University of Alabama at Birmingham, Birmingham, AL USA; 3 Birmingham Veterans Affairs Medical Center, Birmingham, AL USA

## Past

Prior work demonstrating reduced mortality at high-volume hospitals for high-risk cancer surgery has driven increased centralization to referral centers.^1^ The degree to which lower-risk cancer surgery has been centralized is unknown. Although the technical performance of surgery for these “low-risk” tumor types may be straightforward, their oncologic management has become increasingly complex, potentially prompting increased referrals for comprehensive management, even if surgical expertise is available at a lower-volume hospital. The changing landscape of healthcare delivery with a rise in system-based care has prompted more strategic considerations regarding which services to centralize. To inform this, we sought to describe trends in referral for common cancer surgery, including both low- and high-risk oncologic procedures.

## Present

We found an increase in referred care over time for both low- and high-risk procedures.^2^ Specific disease sites with higher odds of referred care relative to breast cancer as a referent group were melanoma, oral cavity tumors, prostate cancer, rectal cancer, and uterine cancer. Sites of definitive management were more often academic and high-volume centers. Patients referred for care were more often from nonmetropolitan areas and travelled further for surgery.

## Future

It is unknown whether, or to what extent, centralization of low-risk cancer surgery benefits patients. Potential unintended consequences of surgical centralization include degradation in local expertise among surgeons working in rural and suburban areas who may not regularly perform certain low-risk procedures, such as sentinel lymph node biopsy for localized melanoma.^3^ Additionally, traveling to a referral center places a greater travel burden on individuals who live in nonmetropolitan areas or those with comorbidities, who as a result may forgo recommended care, even for localized disease.

The rise in health system formation, with over 90% of hospital discharges now originating from system hospitals,^4^ presents an opportunity to think strategically about how to deliver high-quality, cost-effective, integrated care across sites, including intentional centralization and de-centralization of services. To inform these choices, an understanding is needed of the value and cost of centralization of low-risk oncologic procedures within systems, considering both perioperative and oncologic outcomes.^5^ Strategic decentralization of certain lower-risk oncologic procedures within systems may produce equivalent oncologic outcomes while being more patient-centered and less costly for the system.
